# Assessing the importance of thermogenic degassing from the Karoo Large Igneous Province (LIP) in driving Toarcian carbon cycle perturbations

**DOI:** 10.1038/s41467-021-26467-6

**Published:** 2021-10-28

**Authors:** Thea H. Heimdal, Yves Goddéris, Morgan T. Jones, Henrik H. Svensen

**Affiliations:** 1 Centre for Earth Evolution and Dynamics (CEED), University of Oslo, Oslo, Norway; 2 Géosciences-Environnement Toulouse, CNRS-Université Paul Sabatier, Toulouse, France

**Keywords:** Palaeoclimate, Environmental impact, Geochemistry

## Abstract

The emplacement of the Karoo Large Igneous Province (LIP) occurred synchronously with the Toarcian crisis (ca. 183 Ma), which is characterized by major carbon cycle perturbations. A marked increase in the atmospheric concentration of CO_2_ (*p*CO_2_) attests to significant input of carbon, while negative carbon isotope excursions (CIEs) in marine and terrestrial records suggest the involvement of a ^12^C-enriched source. Here we explore the effects of pulsed carbon release from the Karoo LIP on atmospheric *p*CO_2_ and δ^13^C of marine sediments, using the GEOCLIM carbon cycle model. We show that a total of 20,500 Gt C replicates the Toarcian *p*CO_2_ and δ^13^C proxy data, and that thermogenic carbon (δ^13^C of −36 ‰) represents a plausible source for the observed negative CIEs. Importantly, an extremely isotopically depleted carbon source, such as methane clathrates, is not required in order to replicate the negative CIEs. Although exact values of individual degassing pulses represent estimates, we consider our emission scenario realistic as it incorporates the available geological knowledge of the Karoo LIP and a representative framework for Earth system processes during the Toarcian.

## Abstract (web summary)

The emplacement of the Karoo LIP occurred synchronously with the Toarcian crisis, which is characterized by negative carbon isotope excursions. Here the authors use carbon cycle modelling to show that thermogenic carbon released during LIP emplacement represents a plausible source for the negative excursions.

## Introduction

The Toarcian crisis (ca. 183 Ma) is characterized by extinctions in both the marine and terrestrial realm, localized ocean anoxia (The Toarcian Ocean Anoxic Event; T-OAE), global warming and major carbon cycle perturbations^1–15^. The carbon cycle disturbances are evidenced by increases in the atmospheric concentration of CO_2_ (*p*CO_2_;^1–3^) and negative carbon isotope excursions (CIEs) recorded in both carbonate and organic matter in marine and terrestrial archives^4–14^. A prominent negative CIE marks the onset of the T-OAE, and varies in magnitude in both carbonate and organic matter (δ^13^C_carb_ ≈ −0.6 to −6.0‰; δ^13^C_org_ ≈ −0.8 to −8.6‰; see review in ref. ^15^). High-resolution δ^13^C curves from multiple sections show a stepwise character of the excursion, with several abrupt negative shifts making up the full body of the CIE^3,4,6–9,11,12,14–17^.

**Fig. 1:**
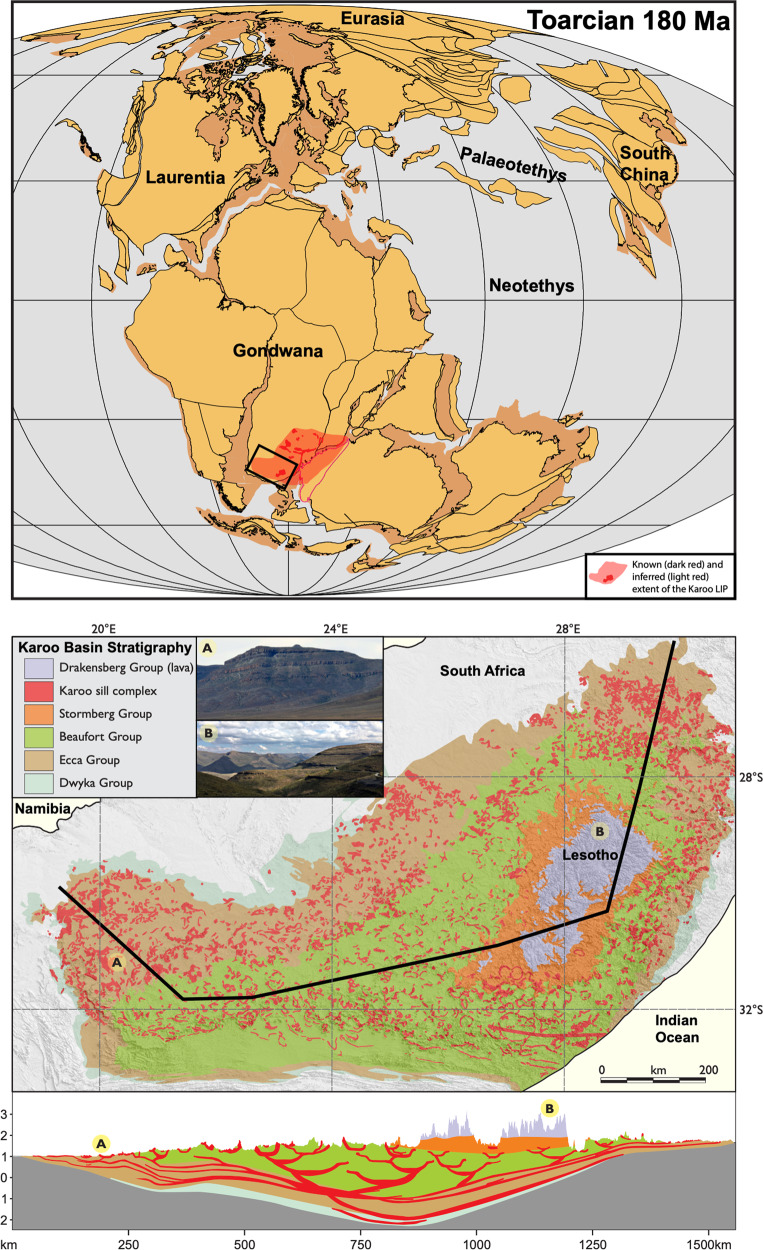
Location and cross section of the Karoo Basin. Top: a plate reconstruction at 180 Ma (modified from ref. ^60^; Fig. 12.2) showing the known and inferred extent of the Karoo Large Igneous Province (LIP)^21,61^. Bottom: a detailed map of the Karoo Basin stratigraphy in South Africa and Lesotho, with a schematic cross section across the province (modified from ref. ^47^). Photo A shows deep laminar sills of the Ecca Group in western South Africa, while photo B shows the continental flood basalt lavas exposed in Lesotho (positions are labelled as A and B on the map and cross section).

**Fig. 2:**
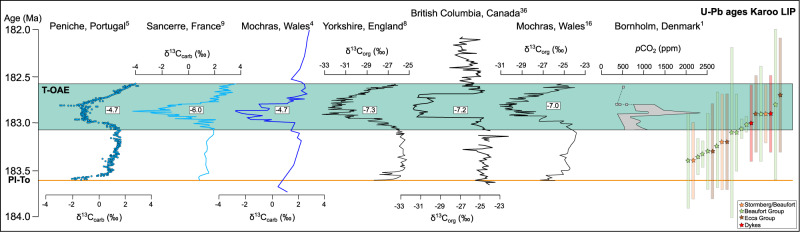
Correlation between Toarcian carbon cycle perturbations and the timing of Karoo intrusions. Timeline of Karoo activity, the Toarcian Ocean Anoxic Event (T-OAE; green box), carbon isotope curves from shallow marine stratigraphic sections and *p*CO_2_ data (including uncertainties) after ref. ^18,19,35^. The age of the Pliensbachian-Toarcian (Pl-To) boundary (orange line) is from ref. ^19^. U-Pb ages of Karoo intrusives are from ref. ^18–21^. U-Pb color codes refer to sill emplacement levels in the Karoo Basin, and corresponds to the colors used in the map and cross section in Fig. 1. Numbers in boxes represent the total negative shift (δ^13^C_max_ − δ^13^C_preT-OAE_) in δ^13^C_org_ and δ^13^C_carb_.

High-precision U-Pb geochronology demonstrates that the Karoo LIP in South Africa and Lesotho (Fig. 1) was emplaced synchronously with the T-OAE negative CIE (Fig. 2; ref. ^18–21^). This has led to the hypothesis that Karoo-derived emissions were responsible for the carbon cycle perturbations (e.g.,^1,7,18–21^,); however, the negative shift in δ^13^C is of such magnitude that mantle-derived carbon alone is unlikely to have been the source^22^. Alternative suggestions include marine methane clathrate dissociation (e.g.,^4,8,22^,), permafrost melting^23^, terrestrial organic matter^24,25^, or thermogenic carbon release generated by sill emplacement into organic-rich sedimentary rocks of the Karoo Basin (e.g.,^1,19,22,26^,).

Volcanic basins, such as the Karoo, are characterized by a pulsed emplacement of sills (e.g.,^27^,), leading to pulsed contact metamorphism and fluid release. Notably, a stepwise decrease in δ^13^C is suggestive of isotopically light carbon supplied in pulses to the ocean-atmosphere system^28^. Field investigations, borehole studies, and numerical modeling suggest thermogenic carbon generation on the order of several thousand gigatons within the Karoo basin^26,29–31^. The presence of thousands of explosion pipes and hydrothermal vent complexes rooted in the contact metamorphic aureoles represent a plausible release mechanism, and attests to rapid emissions of the thermogenic gases^26,32^. Available high-precision U-Pb ages of the sills and thermogenic emission estimates from the Karoo Basin allows to identify detailed and realistic emission scenarios from the Karoo LIP; however, these have not yet been tested by carbon cycle modeling.

Here, we test such scenarios using the GEOCLIM model^33,34^, in order to explore whether pulsed carbon release from the Karoo LIP could explain the full extent of observed Toarcian *p*CO_2_ and δ^13^C changes. The emission scenarios are grounded by U-Pb geochronology, detailed evolution of the Karoo LIP, and estimates of both the mantle-derived and thermogenic carbon from volcanic and contact metamorphic degassing, respectively.

## Results

In order to investigate the consequences of carbon release from the Karoo LIP, it is essential to constrain the timing between the negative CIEs, *p*CO_2_ excursion, and Karoo activity. Following the correlations used here (see Supplementary Note 1;^18,19,35^), the T-OAE lasted for 500 kyr, and overlaps with the full extent of the carbon isotope perturbations (Fig. 2). This corresponds to the onset of the negative CIEs, as well as peak negative values, and the subsequent interval of δ^13^C increase eventually reaching background levels. Reconstructed *p*CO_2_ curves from the Danish Basin show an increase in *p*CO_2_ of up to ~2000 ppm during the T-OAE (including uncertainties; ref. ^1^). Following our correlations, the *p*CO_2_ peak coincides with the peak negative δ^13^C values (Fig. 2). The *p*CO_2_ records do not include absolute time constraints, so the correlation of *p*CO_2_ data with the Karoo activity and various carbon isotope records was based on the extent of the T-OAE (see Supplementary Note 1). Available U-Pb ages of Karoo intrusives demonstrate that the Karoo igneous activity overlaps with the full extent of the Toarcian CIEs and by inference the *p*CO_2_ peak (Fig. 2; ref. ^18–21^).

The Karoo intrusives postdate a ∼1–2‰ negative CIE that occurs around the Pliensbachian-Toarcian (Pl-T) boundary in some sections (e.g.;^5,36^, Fig. 2), excluding a temporal link between Karoo igneous activity and this earlier disruption of the carbon cycle^37^. As this CIE is not systematically recorded, and there is no evidence for *p*CO_2_ increase or Karoo activity at this time, it is not considered in this study. Furthermore, we stress that there are alternative correlations and interpretations for the Toarcian carbon cycle perturbations that differ from those used here, particularly with respect to the duration of the full extent of the T-OAE CIE, which varies from 120 to 2400 kyr (Supplementary Note 1;^4,7,8,18,19,35,38–43^).

The total model run is set to 500 kyr, which corresponds to the total duration of the T-OAE (following the correlations used here; Fig. 2; Supplementary Note 1). Both volcanic and thermogenic processes associated with the Karoo LIP activity are accounted for in the emission scenario. Seven thermogenic carbon pulses (δ^13^C of −36‰; Supplementary Note 1) are released between model time (*t*) = 30 and 262 kyr (pulses #2-8; Table 1; Supplementary Fig. 1), which is constrained by the timing of negative CIEs. The thermogenic carbon pulses vary in magnitude, between 300 and 3800 Gt. The total magnitude of released mantle-derived carbon (with δ^13^C of −5‰) is set to 8000 Gt, following average estimates for the Karoo LIP (e.g., ref. ^22^; Supplementary Note 1). 4500 Gt mantle-derived carbon is released at *t* = 160 (pulse #6; Table 1), while the remaining 3500 Gt C is released as a long-lasting and continuous pulse between *t* = 0 and 262 kyr (pulse #1; Table 1; Supplementary Fig. 1; Supplementary Note 1). Note that carbon pulse #6 represents a mix of both mantle-derived and thermogenic carbon, with a total magnitude of 7000 Gt and a δ^13^C value of −16‰ (Table 1; Supplementary Note 1). Except for the continuous mantle-derived carbon pulse (pulse #1), the carbon releases have durations of 3–22 kyr, which is constrained by the duration of observed carbon cycle perturbations targeted, and in agreement with modeling studies suggesting that the generation of thermogenic fluids occurs on timescales of 100’s to 1000’s years^29,44^.

**Table 1:** Overview and input values for Karoo emission scenario.

Carbon pulse no.	1	2	3	4	5	6	7	8
Magnitude (Gt)								
Volcanic C	3500	N/A	N/A	N/A	N/A	4500	N/A	N/A
Thermogenic C	N/A	300	500	2000	3800	2500	1700	1700
Total	3500	300	500	2000	3800	7000	1700	1700
δ^13^C (‰)								
Volcanic C	-5	N/A	N/A	N/A	N/A	-5	N/A	N/A
Thermogenic C	N/A	−36	−36	−36	−36	−36	−36	−36
Total	−5	−36	−36	−36	−36	−16	−36	−36
Model time t (ky)	0	30	62	82	111	160	210	254
Duration (ky)	262	3	3	8	19	22	11	8
Flux (Gt C/y)	N/A	0,1	0,2	0,3	0,2	0,3	0,2	0,2

N/A, not applicable

**Fig. 3:**
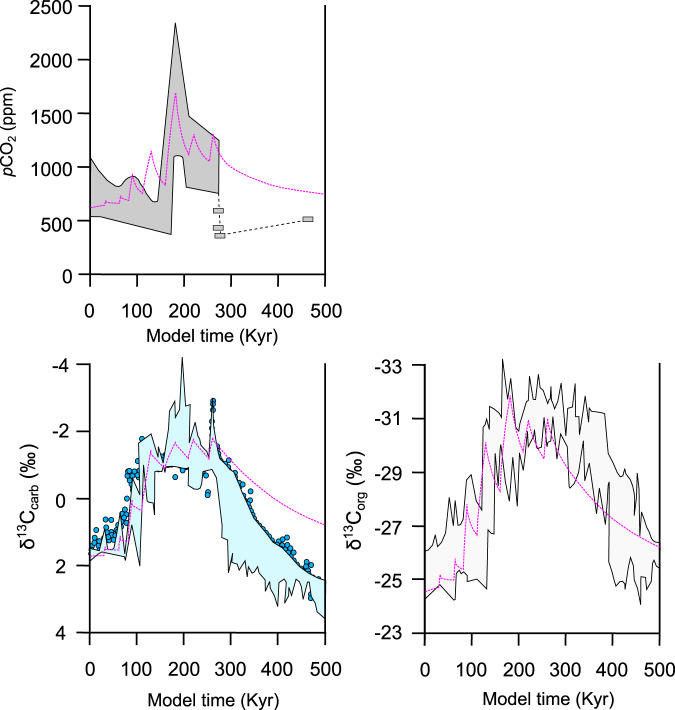
Modeled *p*CO_2_ and δ^13^C curves compared to observed Toarcian data. Model response (pink dashed lines) of δ^13^C_org_ and δ^13^C_carb_ of the shallow ocean (GEOCLIM epicontinental surface reservoir boxes) and atmospheric *p*CO_2_ to a Karoo emission scenario including eight pulses of carbon release (see Table 1; Supplementary Fig. 1). Gray and blue outlines represent the range of observed Toarcian δ^13^C_carb_^4,5,9^, δ^13^C_org_^8,16,36^, and *p*CO_2_ data^1^, which corresponds to the curves presented in Fig. 2.

The pulsed release of a total of 20,500 Gt C leads to a stepwise increase in *p*CO_2_ from ~650 ppm up to a maximum of ~1700 ppm. The modeled *p*CO_2_ curve plots generally within the range of observed data, with the exception of the fourth peak (at *t* = 111 kyr), which overestimates the *p*CO_2_ (Fig. 3). The modeled δ^13^C_carb_ curve decreases in seven steps (between ~0.3 and ~1.8‰), with a total negative shift (δ^13^C_max_ – δ^13^C_preT-OAE_) of ~−4‰. Following the δ^13^C_carb_ data, the modeled δ^13^C_org_ curve also decreases in seven steps (between ~0.5 and ~3.7‰), but with larger magnitudes, as well as a larger a total negative shift of ~−6‰. Both the modeled δ^13^C_carb_ and δ^13^C_org_ curves generally plot within the range of observed data considering both the magnitude and shape of the negative shift (Fig. 3). There are however some exceptions: 1) the modeled δ^13^C_carb_ curve predicts slightly lower δ^13^C values at the end of the model run during the recovery interval of δ^13^C increase, and 2) the modeled δ^13^C_org_ curve does not replicate all of the short-lived negative CIEs making up the prolonged interval of negative δ^13^C values following peak negative values. Note that all observed δ^13^C curves are from shallow marine stratigraphic sections, and is accordingly compared to model δ^13^C data representing a shallow ocean epicontinental sea or shelf environment (i.e., epicontinental surface reservoir boxes; see Methods).

## Discussion

The emission scenario presented here replicates an overall global trend in δ^13^C, including several negative steps, and a total negative shift (δ^13^C_max_ – δ^13^C_preT-OAE_) of ~−4‰ for δ^13^C_carb_ and ~−6‰ for δ^13^C_org_. This is comparable to observed Toarcian carbonate δ^13^C records showing generally lower CIE magnitudes compared to those of organic matter, with mean total CIE values of ~−3 vs. ~−5‰, respectively^15^. Globally, the total magnitude of the T-OAE CIE ranges considerably (up to ~−6 for δ^13^C_carb_ and ~−9‰ for δ^13^C_org_; ref. ^15^), however, the largest excursions are likely exaggerated due to local effects^15,43^. Furthermore, all high-resolution Toarcian carbon isotope curves show a stepwise character of the T-OAE excursion, and a series of at least five prominent negative shifts is recognized in numerous carbonate and organic matter Toarcian sections (e.g., Kaszewy, Mochras, Yorkshire, Peniche and Luxembourg;^12,45^). δ^13^C curves showing one main long-lived T-OAE CIE, or a limited number of negative shifts, have likely failed to record the full extent of the stepped descent due to low sample or stratigraphic resolution. We chose not to take the observed prolonged interval of negative δ^13^C_org_ values following peak negative values into account, because it is uncertain whether or not this represents a global trend. It has been suggested that, due to improved preservation of marine organic matter under oxygen-depleted conditions, artificial Toarcian δ^13^C_org_ excursions have been produced^40^. This could potentially explain why there is an apparent prolonged interval of δ^13^C_org_ CIEs, while δ^13^C_carb_ values instead return to background levels (which is also consistent with the shape of the *p*CO_2_ curve). Although Toarcian δ^13^C curves vary with respect to the magnitude, number, timing, and durations of the negative CIEs, we consider our modeled δ^13^C_carb_ and δ^13^C_org_ curves to be representative for the overall global trend of Toarcian carbon cycle changes.

While the modeled δ^13^C data is compared to several observed δ^13^C curves, available reconstructed Toarcian *p*CO_2_ curves are scarce. As seen in Fig. 3, the modeled *p*CO_2_ curve generally plots within the range of observed Toarcian *p*CO_2_ data from ref. ^1^, but potential target *p*CO_2_ values represent quite a wide range due to data points with large error bars. Consequently, the magnitude of released carbon could vary greatly and still replicate the observed *p*CO_2_ data within the range of uncertainties. However, by respecting both observed pCO_2_ and δ^13^C curves simultaneously, the possible magnitude of carbon release is narrowed down considerably. Furthermore, based on Toarcian seawater pH (ref. ^2^) and Δ13C data (δ^13^C_terrigenic_ – δ^13^C_marine_; ref. ^3^), a *p*CO_2_ increase from ~800–850 ppm to ~1750–1800 ppm has been calculated (when assuming a release of thermogenic carbon with δ^13^C of −40 to −30‰ for the latter; ref. ^3^). As seen in Fig. 3, an increase of this magnitude (i.e., 800–850 to 1750–1800 ppm) is in very close agreement with the modeled *p*CO_2_ data. The overestimation of *p*CO_2_ at *t* = 111 kyr shown by the modeled curve (Fig. 3) is likely the result of the low sample resolution of the observed data, as it is very unlikely that a shift in δ^13^C of up to 4‰ is not reflected by a change in *p*CO_2_. Therefore, we consider the modeled *p*CO_2_ curve presented here to be realistic as it reflects Toarcian *p*CO_2_ estimates constrained by several methods.

According to our emission scenario, a total release of 20,500 Gt carbon replicates the observed Toarcian *p*CO_2_ and δ^13^C data. This number includes 12,500 Gt thermogenic carbon, which is within the range of thermogenic carbon generation estimations from the Karoo Basin (~7500–23,000 Gt C; ref. ^26,29–31^). The ratio of trapped versus released thermogenic gases from volcanic basins is poorly constrained, although 100% degassing from intrusive LIP components is considered unrealistic (e.g., ^46^,). Consequently, the magnitude of released carbon must be lower than the magnitude of carbon generated. The lowermost thermogenic carbon generation estimates are lower than the release value constrained by our modeling. However, these numbers are likely significantly underestimated as they have been upscaled based on a much smaller area of affected host rock compared to that of ref. ^31^, and do not include devolatilization of Ecca coals or Beaufort, Dwyka and Stormberg organic-bearing rocks in the Karoo strata (see Fig. 1). According to ref. ^31^, an estimated 72 % of the generated carbon gases in the Karoo Basin were released, which corresponds to ~17,000 Gt C. We therefore consider 12,500 Gt to represent a realistic value of cumulative thermogenic carbon release from the Karoo Basin.

Considering the TOC content and sill distribution in the Karoo Basin, sill emplacement into Ecca and Beaufort shales volatilized significantly more carbon-bearing gases compared to sill emplacement at other stratigraphic levels. Within the Ecca and Beaufort series, sills are abundant and can be traced almost continuously with depth, as well as between the basin margins (Fig. 1; ref. ^47^). In contrast, sills are rare in the Stormberg Group, while devolatilization of Dwyka sedimentary rocks was likely restricted to the upper parts of the series based on the distribution of the sills. The Ecca shales have the highest TOC contents, reaching locally as much as 18 wt.%, while maximum values within the Stormberg, Beaufort and Dwyka Group sedimentary rocks range from 3 to 6 wt.%^48–53^. Although the TOC contents are overall lower compared to the Ecca Group, the presence of closely spaced large sills (up to 270 m thick; ref. ^47^) in the Beaufort Group with TOC up to ~6 wt.%^52^ suggests significant gas generation potential at these stratigraphic levels as well. Sill thicknesses and vertical spacing are important factors influencing the gas generation potential; gas generation can increase by up to ~35 % for more closely spaced sills, compared to separate sills emplaced into the same host-rock^27^.

According to our emission scenario, the magnitudes of the individual thermogenic carbon pulses vary throughout the model run (Table 1), which is constrained by the magnitudes of the observed carbon cycle perturbations targeted. The available U-Pb data cannot be used to directly assess the magnitude of the individual degassing pulses as 1) there is no systematic correlation of sill age with stratigraphic emplacement level, and 2) they lack the sufficient resolution (large error bars) to match that of the stepped δ^13^C curves. Importantly, however, the U-Pb geochronology links sill emplacements within organic-rich sedimentary rocks (i.e., Ecca and Beaufort) to the entire interval of Toarcian carbon cycle perturbations (Fig. 2). Furthermore, the proposed carbon emission fluxes of 0.2 to 0.3 Gt C yr^−1^ (Table 1) corresponds to those estimated for other LIPs (e.g., NAIP and CAMP; 0.1–0.5 Gt C yr^−1^; ref. ^54,55^). We therefore consider the magnitudes of the individual pulsed thermogenic carbon releases to represent realistic values.

Realistic temporal and volumetric estimates of Karoo LIP volatile emissions can explain the carbon cycle perturbations observed in the Toarcian. Pulsed sill intrusions and contact metamorphism of organic matter-bearing sedimentary rocks in the Karoo Basin represents a significant source of ^13^C-depleted carbon, which can explain the observed negative CIEs. As the lowest δ^13^C value of the released thermogenic carbon in our model is −36‰, an extremely isotopically depleted carbon source (e.g., methane clathrates; −60‰; ref. ^22^) is not a required component (see also Supplementary Fig. 2). High-precision U-Pb geochronology links sill emplacements within the organic-rich strata of the Karoo Basin to the negative CIEs and the abrupt *p*CO_2_ peak. Although the exact values for the different degassing pulses represent estimates, we consider our emission scenario realistic as it incorporates the available geological knowledge of the Karoo LIP and a representative framework for Earth system processes during the Toarcian.

## Methods

### Model description

GEOCLIM couples the 10-box model COMBINE^56^, describing different geochemical cycles (e.g., C, P, O_2_, alkalinity, Sr) and their associated isotopic cycles (δ^13^C, ^87^Sr/^86^Sr), to the 3D FOAM general circulation climate model^57^. COMBINE consists of one atmospheric box and nine ocean boxes, including 1) an epicontinental surface reservoir divided in two boxes (surface box up to 100 m depth; deep box from 100 to 200 m depth), 2) three mid-latitude ocean boxes (surface, thermocline, and deep), and 3) two polar oceans both divided in two boxes (surface and deep). A full description of the GEOCLIM model can be found in refs. ^34^ and ^56^.

Boundary conditions for the simulations presented here include a Jurassic (180 Ma) paleogeography, which is derived from a synthesis of paleomagnetic data, hot spot tracks and geological constraints^34,58^. The background volcanic degassing rate was set to 4 × 10^12^ mol/yr so that the *p*CO_2_ equals ~ 650 ppm at t = 0 (prior to any Karoo degassing), which represents an average value of observed *p*CO_2_ data (including uncertainties; ref. ^1^).

GEOCLIM calculates the δ^13^C of the various carbon species (e.g., H_2_CO_3_, POC; particulate organic carbon) in each reservoir/box (e.g., epicontinental surface box). The isotopic fractionation between H_2_CO_3_* (H_2_CO_3_ + CO_2_; Supplementary Note 1) and POC is set to depend on the calculated H_2_CO_3_* concentration at each timestep for each oceanic reservoir. As shown in Supplementary Fig. 3a, the fractionation between H_2_CO_3_* and the POC increases during the interval of carbon injections, but then decreases towards “background values”. A decrease in fractionation reduces the difference between the δ^13^C of carbonate and POC, which allows the δ^13^C_org_ to increase faster compared to δ^13^C_carb_^59^. This could potentially explain the apparent decoupling between the δ^13^C_org_ and δ^13^C_carb_ modeled curves (Fig. 3; Supplementary Fig. 3b); while the modeled δ^13^C_org_ curve recovers faster and reach observed values during the recovery interval at the end of the model run, the modeled δ^13^C carb curve predicts slightly lower δ^13^C values compared to observed values.

## Supplementary information

Supplementary Information

## Data Availability

All data used for this paper is available in the main text and in the Supplementary Information (Supplementary Note 1, Supplementary Figs. 1–3 and Supplementary References).

## Code availability

The GEOCLIM source code is available upon request to the first author.
