# Supplementary material for: Assessing the importance of thermogenic degassing from the Karoo Large Igneous Province (LIP) in driving Toarcian carbon cycle perturbations
Source: Nat Commun. 2021 Oct 28;12:6221. doi: 10.1038/s41467-021-26467-6 (PMC8553747; doi:10.1038/s41467-021-26467-6)
Supplement: Supplementary file 1 — Supplementary Information [file 41467_2021_26467_MOESM1_ESM.pdf]

Supplementary Note 1

Assessing the importance of thermogenic degassing from the Karoo Large Igneous Province (LIP) in driving Toarcian carbon cycle perturbations

Thea H. Heimdal[1*], Yves Goddéris[2], Morgan T. Jones[1] & Henrik. H. Svensen[1]

[1]Centre for Earth Evolution and Dynamics (CEED), University of Oslo, Oslo, Norway.

[2]Géosciences-Environnement Toulouse, CNRS-Université Paul Sabatier, Toulouse, France.

*Corresponding author: Thea Hatlen Heimdal, Centre for Earth Evolution and Dynamics (CEED), University of Oslo, PO Box 1028, Blindern, NO-0315 Oslo, Norway, +47 41548103, t.h.heimdal@geo.uio.no.

The estimated $\delta^{13}C$ of the emitted carbon

Two types of carbon pools were considered in the emission scenario (mantle and organic matter; Supplementary Fig 1), and consequently, the emitted carbon gases from these pools will have different $\delta^{13}C$ values. Mantle-derived carbon was given the generally accepted $\delta^{13}C$ value of -5 ‰ (e.g., 1-3). Carbon is released as both CH_4 and CO_2 during contact metamorphism of organic matter-bearing rocks (4). Early thermogenic gases generated in shales are ^{12}C-enriched, but as the source rocks become increasingly thermally mature, the $\delta^{13}C$ of the released carbon increases (5). If all organic matter is lost from the host rocks during contact metamorphism, the total $\delta^{13}C$ value of the released carbon should correspond to the bulk pre-metamorphic value (4). This is only the case for the innermost "hornfels zone" of the contact aureole close to the sill contact (6-8). The $\delta^{13}C$ of the released thermogenic carbon in

the emission scenario (i.e., $CO_2 + CH_4$) was based on a simple mass balance calculation, where *M* equals magnitude:

$$\delta^{13}C_{org} = \frac{(\delta^{13}C_{CH_4} \times MC_{CH_4}) + (\delta^{13}C_{CO_2} \times MC_{CO_2})}{MC_{org}}$$

The parameter $\delta^{13}C_{CO2}$ was given a value of -22 ‰, based on measured $\delta^{13}C$ values of organic matter in the Ecca Group shale in the Karoo Basin (-18 to -25‰; ref. 9-11), while $\delta^{13}C_{CH4}$ was set to -50 ‰, which represents an average value of measured $\delta^{13}C$ values for CH_4 (i.e., -20 to -75 ‰; ref. 5, 12-13). The ratio of released CH_4 versus CO_2 from volcanic basins is poorly constrained, so we assume a ratio of 1:1. Based on the mass balance calculation presented above, this yields a total $\delta^{13}C_{org}$ value of -36‰.

For carbon pulse #6, which includes both mantle-derived and thermogenic carbon, the total $\delta^{13}C$ value was determined based on a simple mass balance calculation, where *M* equals magnitude:

$$\delta^{13}pulse6 = \frac{(\delta^{13}C_{org}\ MC_{org}) + (\delta^{13}C_{volc}\ MC_{volc})}{MC_{pulseX}}$$

The parameters $\delta^{13}C_{org}$ (organic-derived thermogenic carbon), and $\delta^{13}C_{volc}$ (mantle-derived carbon) equal -36 and -5 ‰, respectively, following the discussion above.

Alternative emission scenario

As discussed above, the value of -36 ‰ for the thermogenic carbon represent an estimate. As a limit case, we set up an alternative emission scenario (Supplementary Table 1; Supplementary

Fig. 2) where the thermogenic carbon was given a $\delta^{13}C$ value corresponding to that of the organic matter in the Ecca shale (i.e., -22 ‰). This limit case represents therefore an upper boundary for the $\delta^{13}C$ signature of the released thermogenic carbon. The timing and duration of all carbon pulses, as well as the magnitude of mantle-derived carbon release, are identical to those of the emission scenario presented in the main text. This upper boundary limit case includes three runs, where the magnitude of released carbon is 1) identical to that in the main text (run A), 2) 1,450 Gt higher compared to that of the main text (run B), and 2,900 Gt higher compared to that of the main text (run C).

As shown by Supplementary Figure 2, more carbon is needed in order to replicate the negative CIEs. The model results for run B and C generally plot within the range of observed Toarcian proxy data, but slightly overestimates some of the pCO_2 data, and do not replicate the largest $\delta^{13}C_{carb}$ CIEs. The maximum difference in magnitude of carbon released in this alternative emission scenario is only 2,900 Gt C, which includes up to 15,400 Gt thermogenic carbon. Releasing thermogenic carbon of this magnitude is still in agreement with model results from the Karoo Basin, as discussed in the main text (up to ~ 17,000 Gt C; ref. 14). Although a $\delta^{13}C$ value of -22 ‰ is likely on the higher end, the main conclusion from this alternate setup is that the release of carbon which represent a range in potential $\delta^{13}C$ values of thermogenic carbon, will not significantly affect the emission scenario nor the interpretation of the results. Furthermore, these results strengthen the case that an extremely isotopically depleted carbon source such as methane clathrates ($\delta^{13}C$ of ca. -60 ‰; e.g., ref. 15) is not a required component in order to explain the negative CIEs.

Estimations of mantle-derived carbon release from the Karoo LIP

The magnitude of released mantle-derived carbon associated with the Karoo LIP emplacement is poorly constrained. By assuming a degassing rate of ~ 4.4 Mt C/km^3 and a pre-erosional Karoo LIP volume of 2.0 x 10^6 km^3 (ref. 16), ref. 15 calculated a total release of ~ 8,700 Gt volcanic C, which they considered to be a high-end estimate. Estimating pre-erosional volumes of LIPs, particularly those emplaced at tropical latitudes, is challenging because lava flows and exposed intrusives have often been significantly eroded, which is also the case for Karoo. For example, ref. 17 suggested a pre-erosional LIP volume of > 2.5 x 10^6 km^3, which is notably larger than the volume considered by ref. 15. Furthermore, estimating pre-eruptive carbon magma concentrations is challenging as CO_2 is quite insoluble in magmas and exsolution occurs relatively deep in the crust. Ref. 18 considers a degassing rate of 3.6 Mt C/km^3 to be on the higher end (corresponds to 100% degassing of 0.5 wt.% CO_2), which is lower than the value considered by ref. 15 (i.e., ~ 4.4 Mt C/km^3). If we consider a possible range in the degassing rate of 3.6 to 4.4 Mt C/km^3 and pre-erosional volume of 2.0 to 2.5 x 10^6 km^3, the potential volcanic carbon release ranges from 7,200 to 11,000 Gt. Given the uncertainties involved when estimating both pre-erosional LIP volumes and pre-eruptive CO_2 magma concentrations, volcanic carbon release estimates should be considered with caution.

The exact timing of volcanic degassing is currently unknown as U-Pb ages of Karoo lavas do not exist. There is no evidence pointing toward the release of several short-lived pulses of mantle-derived carbon, or a decoupling between volcanic and thermogenic degassing. Therefore, for simplicity, mantle-derived carbon was released as a continuous pulse in our emission scenario. In addition, an assumption of an additional pulse of mantle-derived release was made based on available data; a major increase in pCO_2 (at t = 160 kyr) attest to significant carbon release at this time. In our emission scenario we tested therefore a combination of continuous and pulsed mantle-derived carbon release, however the end-member scenarios

(purely pulsed vs. purely continuous release) cannot be ruled out. Improved high-precision U-Pb geochronology could potentially better constrain the exact timing and nature of the Karoo LIP mantle-derived carbon release.

Correlation of Toarcian $p\mathrm{CO_2}$ and δ^{13}C records

Ref. 19-20 present correlations of δ^{13}C curves that include absolute age constrains. As several Karoo intrusives have been dated by high precision U-Pb geochronology, this allows for comparison between carbon cycle changes and Karoo igneous activity. The correlation from ref. 20 includes a compilation of $\delta^{13}C_{carb}$ curves (from ref. 21), and age constraints for the T-OAE, based on different astronomical calibrations of the early Toarcian (900 kyr: ref. 22 and 500 kyr: ref. 23). The $\delta^{13}C_{carb}$ compilation seems however to be somewhat simplified. For example, the distinct negative steps of the CIE presented in the original data set in ref. 24 are not apparent. Ref. 19 presents both $\delta^{13}C_{org}$ and $\delta^{13}C_{carb}$ curves from different sections worldwide including ammonite zones, and infer a ~ 300 kyr duration for the Toarcian carbon cycle disturbances. With closer inspection, the curve from British Columbia shows considerably lower $\delta^{13}C_{org}$ values before and after the T-OAE compared to the original data set (25). Furthermore, the curve from Peru (26) does not include negative excursions, and the curve from Argentina (27-28) shows anomalously high $\delta^{13}C_{org}$ values before and after the T-OAE (~ -23 ‰) compared to the majority of published $\delta^{13}C_{org}$ data.

Based on the absolute ages, we combined the timelines by ref. 19-20. The duration of the T-OAE is generally estimated to between 300 and 500 kyr (19, 23, 29-32), although much shorter (120 kyr; ref. 33) and longer durations (900 kyr; ref. 22; up to 2,400 kyr; ref. 34) have also been suggested. We chose the 500 kyr calibration for the T-OAE, and included the δ^{13}C curves from ref. 23 in the combined timeline ($\delta^{13}C_{carb}$ curves: ref. 24, 35; $\delta^{13}C_{org}$ curve: ref. 29). Note that we chose not to correlate these δ^{13}C curves beyond the end of the T-OAE, due

to uncertain age constraints. The age of the Pliensbachian-Toarcian (Pl-To) boundary (orange line) is from ref. 20, which differs from the age presented in ref. 19, however these two ages overlap within uncertainties. Additional $\delta^{13}C_{org}$ and $\delta^{13}C_{carb}$ curves from British Columbia and Wales were included based on the ammonite zones from ref. 19, but using the original data sets from ref. 25 and 36. Finally, a $\delta^{13}C_{org}$ curve from Mochras (37) was added based on its correlation to the Yorkshire curve (29) and the Pl-To boundary.

Targeting observed pCO_2 and $\delta^{13}C$ values simultaneously considerably constrains the magnitude of carbon potentially responsible for the observed perturbations, so it is crucial to include both proxies in the correlations. Reconstructed pCO_2 records from the Danish Basin (based on stomatal index/density of Coniferales/Ginkgoales; ref. 38) show a significant pCO_2 increase associated with the T-OAE, however they do not include absolute age constraints. Ref. 38 compared these pCO_2 records to a carbon isotope record from the same sedimentary succession (Sorthat Formation; ref. 36). The carbon isotope record shows a distinct negative CIE, which is divided into two parts. ´Phase A` marks the onset of the CIE and coincides with the onset of the T-OAE, while ´phase B` includes peak negative $\delta^{13}C$ values before reaching background values at the very end of the phase. We therefore assume that the extent of phase A + B represents the extent of the T-OAE. Since the T-OAE is defined in the combined $\delta^{13}C$ correlations described above, the pCO_2 data within the time frame of the T-OAE was included in these correlations. They show that the pCO_2 values range between ~ 500 and 1,000 ppm at the onset of the T-OAE and remains relatively constant, before an abrupt and significant peak of up to ~ 2,000 ppm (including uncertainties) occurs coincident with the peak negative $\delta^{13}C$ values (Fig. 2).

Isotopic fractionation between H_2CO_3* and organic carbon

In the oceans, aqueous (solvated) CO_2 and carbonic acid (H_2CO_3) exist through an equilibrium reaction. Photosynthesis favors CO_2, which is the more abundant species compared to H_2CO_3. Following common conventions, GEOCLIM assumes however that all dissolved CO_2 is in the form of H_2CO_3 (i.e., H_2CO_3*), and adapts the equilibrium constant between atmospheric CO_2 and H_2CO_3* (Henry´s law), and between HCO_3^- and H_2CO_3* accordingly (39). The isotopic fractionation during photosynthesis is thus calculated between H_2CO_3* and organic carbon (40).

Supplementary Figure 1

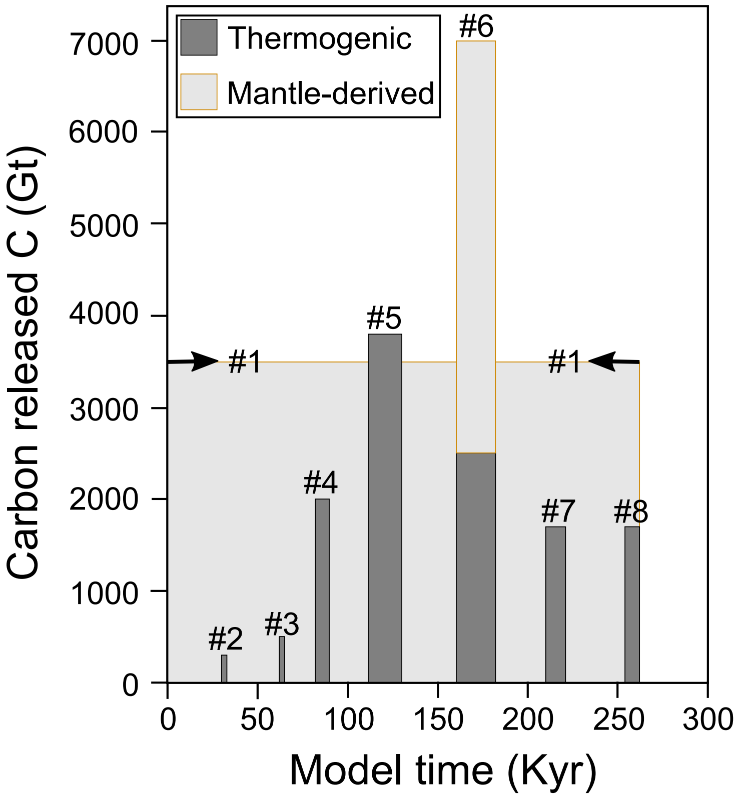

Overview of the emission scenario presented in the main text, including eight carbon pulses (#1-8 in figure). Carbon pulse #1 includes a continuous release of mantle-derived carbon, #6 represents a mixture of thermogenic (light grey color) and mangle-derived carbon (dark-grey

color), while the remaining pulses are thermogenic carbon. The y-axis represents the magnitude of carbon released, while the x-axis corresponds to the GEOCLIM model time.

Supplementary Figure 2

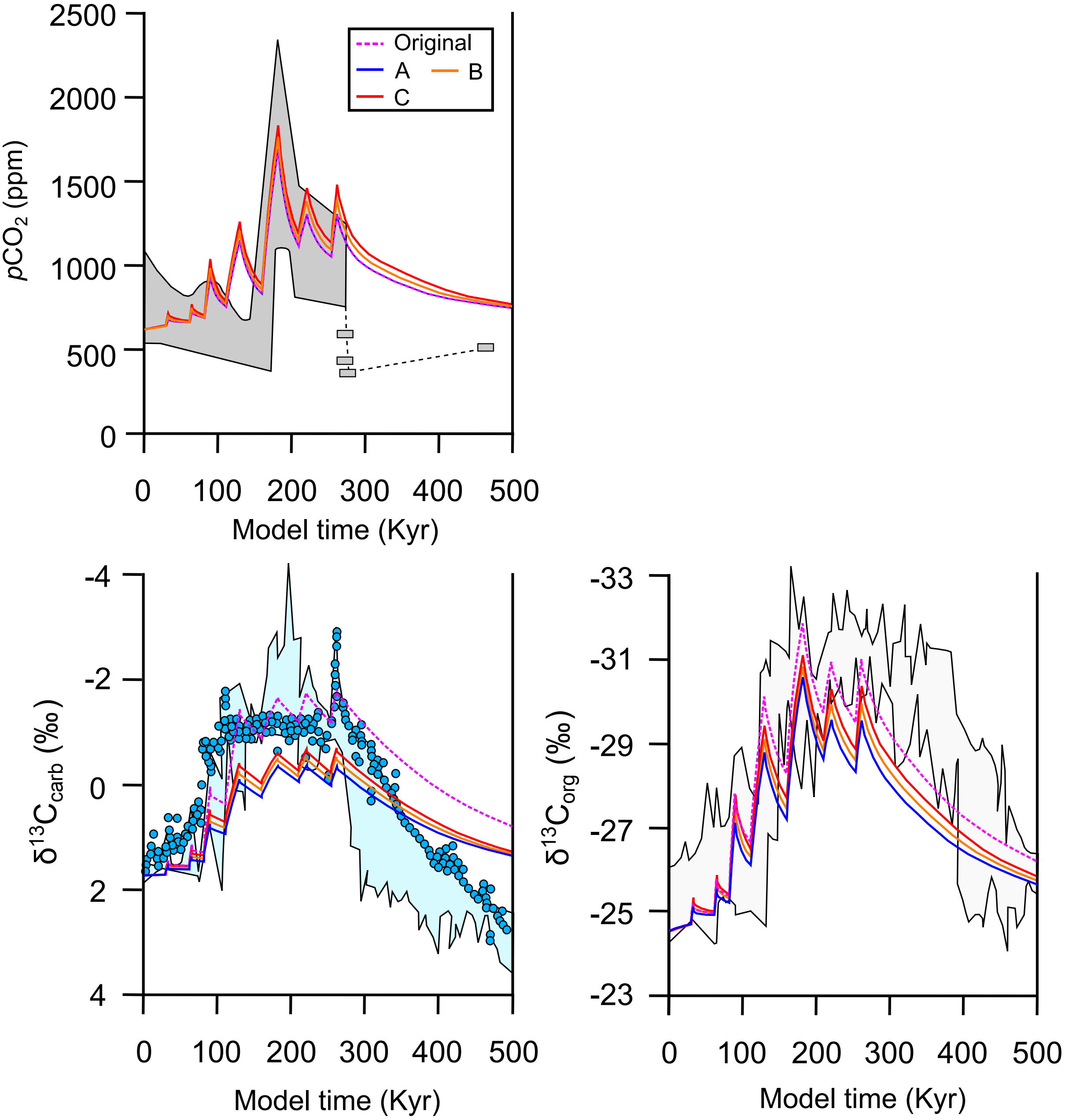

Model response of $\delta^{13}C_{org}$ and $\delta^{13}C_{carb}$ of the shallow ocean (GEOCLIM epicontinental surface reservoir boxes) and atmospheric pCO_2 to the alternative limit case emission scenario (blue, red and orange lines; runs A-C; see Supplementary Table 1). The emission scenario presented in the main text is added for comparison (pink dashed line named "original"). Gray and blue

outlines represent the range of observed Toarcian $\delta^{13}C_{carb}$ (24, 35-36), $\delta^{13}C_{org}$ (25, 29, 37), and pCO_2 data (38), which corresponds to the curves presented in Figure 2 and Figure 3 in the main text.

Supplementary Figure 3

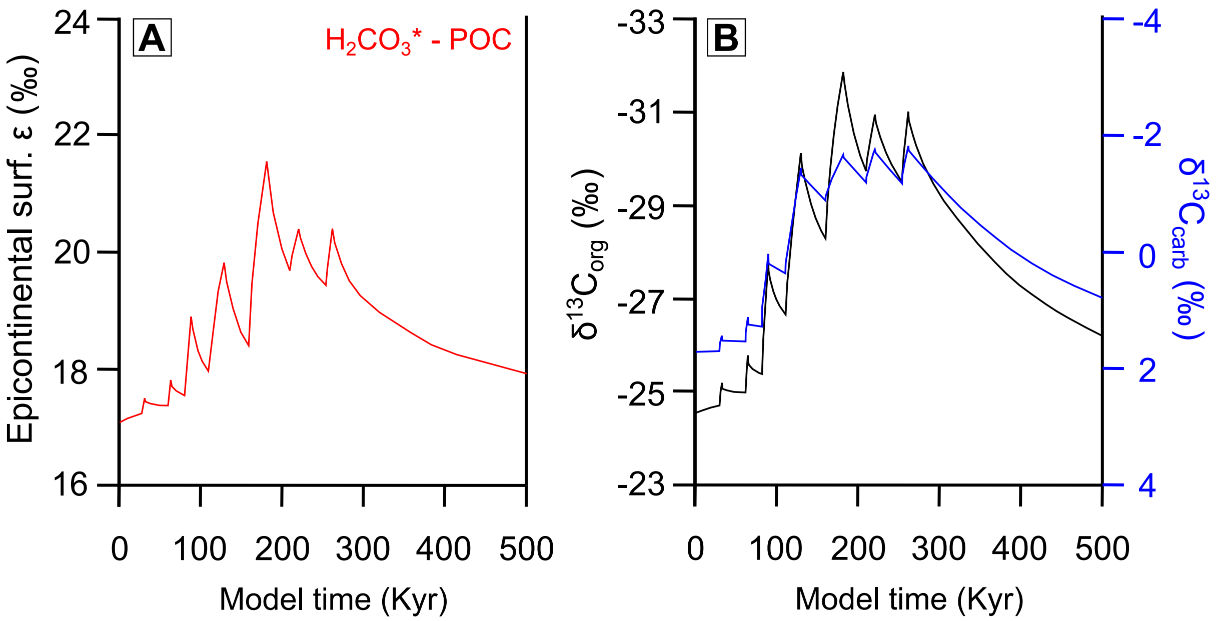

A: The evolution of the isotopic fractionation between H_2CO_3* and particulate organic carbon (POC) in the GEOCLIM epicontinental surface reservoir during the emission scenario presented in the main text (Table 1). **B**: Modeled $\delta^{13}C_{org}$ (black line) and $\delta^{13}C_{carb}$ (blue line) curves (same as in Figure 3) show a faster recovery for $\delta^{13}C_{org}$ towards the end of the model run.

Supplementary Table 1

Overview and input values for the alternative limit case emission scenario

**Carbon pulse no.**	**1**	**2**	**3**	**4**	**5**	**6**	**7**	**8**	
**Magnitude (Gt)**									
**CASE A**									
Volcanic C	3500	N/A	N/A	N/A	N/A	4500	N/A	N/A	Total
Thermogenic C	N/A	300	500	2000	3800	2500	1700	1700	12500
Total	3500	300	500	2000	3800	7000	1700	1700	20500
**CASE B**									
Volcanic C	3500	N/A	N/A	N/A	N/A	4500	N/A	N/A	Total
Thermogenic C	N/A	400	600	2250	4050	2750	1950	1950	13950
Total	3500	400	600	2250	4050	7250	1950	1950	21950
**CASE C**									
Volcanic C	3500	N/A	N/A	N/A	N/A	4500	N/A	N/A	Total
Thermogenic C	N/A	500	700	2500	4300	3000	2200	2200	15400
Total	3500	500	700	2500	4300	7500	2200	2200	23400
**δ^{13}C (‰)**	-5	-22	-22	-22	-22	-12	-22	-22	
Model time t (ky)	0	30	62	82	111	160	210	254	
Duration (ky)	262	3	3	8	19	22	11	8	

N/A, not applicable

References

1. Shirey, S. B., *et al*. Diamonds and the geology of mantle carbon. *Reviews in Mineralogy & Geochemistry*, 75, 355-421 (2013).

2. Cartigny, P., Palot, M., Thomassot, E., & Harris, J. W. Diamond formation: A stable isotope perspective. *Annual Review of Earth and Planetary Science Letters*, 42, 699-732 (2014).

3. Gales, E., Black, B., & Elkins-Tanton. Carbonatites as a record of the carbon isotope composition of large igneous province outgassing. *Earth and Planetary Science Letters*, 535, 116076 (2020).

4. Aarnes, I., Svensen, H., Connolly, J. A., & Podladchikov, Y. Y. How contact metamorphism can trigger global climate changes: Modeling gas generation around igneous sills in sedimentary basins. *Geochimica et Cosmochimica Acta*, 74(24), 7179-7195 (2010).

5. Milkov, A. V., & Etiope, G. Revised genetic diagrams for natural gases based on a global dataset of >20,000 samples. *Organic Geochemistry*, 125, 109-120 (2018).

6. Svensen, H., Planke, S., Chevallier, L., Malthe-Sørensen, A., Corfu, F., & Jamtveit, B. Hydrothermal venting of greenhouse gases triggering Early Jurassic global warming. *Earth and Planetary Science Letters*, 256, 554-566 (2007).

7. Aarnes, I., Svensen, H., Polteau, S., & Planke, S. Contact meatmorphic devolatilizaton of shales in the Karoo Basin, South Africa, and the effects of multiple sill intrusions. *Chemical Geology*, 281, 181-194 (2011).

8. Svensen, H. H. *et al.* Understanding thermogenic degassing in Large Igneous Provinces: Inferences from the geological and statistical characteristics of breccia pipes in the western parts of the Karoo Basin. Geological Society of America Books, Special Paper, 544 (2020).

9. Faure, K., & Cole, D. Geochemical evidence for lacustrine microbial blooms in the vast Permian Main Karoo, Paraná, Falkland Islands and Huab basins of southwestern Gondwana. *Palaeogeography, Palaeoclimatology, Palaeoecology*, 152, 189-213 (1999).

10. Henning, A., van der Westhuizen, W. A., de Bruiyn, H., & Beukws, G. J. Hydrothermal Cu-Ni-Au-Ag mineralization in a granodiorite sill north of Cradock, Republic of South Africa. *Mineralium Deposita*, 32, 410-418 (1997).

11. de Wit, M. J. Organic carbon isotope stratigraphy of the Karoo Supergroup in *Origin and Evolution of the Cape Mountains and Karoo Basin* (eds. Linol, B., & de Wit, M. J.), 169-180 (Regional Geology Reviews, 2016).

12. Schoell, M. The hydrogen and carbon isotopic composition of methane from natural gases of various origins. *Geochimica et Cosmochimica Acta*, 44(5), 649-661 (1980).

13. Andresen, B., Throndsen, T., Råheim, A., & Bolstad, J. A comparison of pyrolysis products with models for natural gas generation. *Chemical Geology*, 126, 161-280 (1995).

14. Galerne, C. Y., & Hasenclever, J. Distinct degassing pulses during magma invasion in the stratified Karoo Basin – new insights from hydrothermal fluid flow modeling. *Geochemistry, Geophysics, Geosystems*, 20, 2955-2984 (2019).

15. Beerling, D. J., & Brentnall, S. J. Numerical evaluation of mechanisms driving Early Jurassic changes in global carbon cycling. *Geology*, 35(3), 247-250 (2007).

16. Jourdan, F., Féraud, G., Bertrand, H., Watkeys, M. K., Renne, P. R. The $^{40}Ar/^{39}Ar$ ages of the sill complex of the Karoo large igneous province: implications for the Pliensbachian–Toarcian climate change. *Geochemistry, Geophysics, Geosystems*, 9, 1–20 (2008).

17. Courtillot, V. E., & Renne, P. R. On the ages of flood basalt events. *Comptes Rendus Geoscience*, 335, 113–140 (2003).

18. Self, S., Thordarson, T., & Widdowson, M. Gas Fluxes from Flood Basalt Eruptions. *Elements*, 1, 283-287 (2005).

19. Sell, B. *et al*. Evaluating the temporal link between the Karoo LIP and climatic–biologic events of the Toarcian Stage with high-precision U–Pb geochronology. *Earth and Planetary Science Letters*, 408, 48–56 (2014).

20. Burgess, S. D., Bowring, S. A., Fleming, T. H., & Elliot, D. H. High-precision geochronology links the Ferrar large igneous province with early-Jurassic ocean anoxia and biotic crisis. *Earth and Planetary Science Letters*, 415, 90-99 (2015).

21. Pittet, B., Suan, G., Lenoir, F., Duarte, L. V., Mattioli, E. Carbon isotope evidence for sedimentary discontinuities in the lower Toarcian of the Lusitanian Basin (Portugal): sea level change at the onset of the Oceanic Anoxic Event. *Sediment. Geol*, 303, 1–14 (2014).

22. Suan, G., Pittet, B., Bour, I., Mattioli, E., Duarte, L.V., Mailliot, S. Duration of the Early Toarcian carbon isotope excursion deduced from spectral analysis: consequence for its possible causes. *Earth and Planetary Science Letters*, 267, 666–679 (2008).

23. Boulila, S. *et al*. Astronomical calibration of the Toarcian Stage: implications for sequence stratigraphy and duration of the early Toarcian OAE. *Earth and Planetary Science Letters*, 386, 98–111 (2014).

24. Hermoso, M., Callonnec, L.L., Minoletti, F., Renard, M., Hesselbo, S. P. Expression of the Early Toarcian negative carbon-isotope excursion in separated carbonate microfractions (Jurassic, Paris Basin). *Earth and Planetary Science Letters*, 277, 194–203 (2009).

25. Caruthers, A. H., Grocke, D.R., Smith, P. L. The significance of an Early Jurassic (Toarcian) carbon-isotope excursion in Haida Gwaii (Queen Charlotte Islands), British Columbia, Canada. *Earth and Planetary Science Letters*, 307, 19–26 (2011).

26. Guex, J., Bartolini, A., Spangenberg, J., Vicente, J.-C., Schaltegger U. Ammonoid multi-extinction crises during the Late Pliensbachian-Toarcian and carbon cycle instabilities. *Solid Earth Discussions*, 4, 1205-1228 (2012).

27. Mazzini, A., Svensen, H., Leanza, H. A., Corfu, F., Planke, S. Early Jurassic shale chemostratigraphy and U–Pb ages from the Neuquen Basin (Argentina): implications for the Toarcian Oceanic Anoxic Event. *Earth and Planetary Science Letters*, 297, 633–645 (2010).

28. Al-Suwaidi, A. H. *et al*. First record of the Early Toarcian Oceanic Anoxic Event from the Southern Hemisphere, Neuquen Basin, Argentina. *Journal of the Geological Society*, 167, 633–636 (2010).

29. Kemp, D.B., Coe, A. L., Cohen, A. S., Schwark, L. Astronomical pacing of methane release in the Early Jurassic period. *Nature*, 437, 396–399 (2005).

30. Kemp, D. B., Coe, A.L., Cohen, A. S., Weedon, G. P. Astronomical forcing and chronology of the early Toarcian (Early Jurassic) oceanic anoxic event in Yorkshire, UK. *Paleoceanography*, 26, 1–17 (2011).

31. Krencker, F.-N., Bodin, S., Suan, G., Kabiri, L., & Immenhauser, A. Assessing the duration and possible causes of the earliest Toarcian carbon isotopic excursion. *EGU General Assembly Conference Abstracts*, 4970 (2013).

32. Suan, G., van de Schootbrugge, B., Adatte, T., Fiebig, J., Oschmann, W. Calibrating the magnitude of the Toarcian carbon cycle perturbation. *Paleoceanography*, 30, 495–509 (2015).

33. Clémence, M. E. *et al*. Micropalaeontologic, geochemical and cyclostratigraphic approach for the timing of the early Toarcian oceanic anoxic event in the Paris Basin (GPF-Sancerre borehole), *Volumnia Jurassica*, 4, 154-156 (2006).

34. McArthur, J. M., Donovan, T. D., Thirlwall, M. F., Fouke, B.M, & Mattey, D. Strontium isotope profile of the early Toarcian (Jurassic) oceanic anoxic event, the duration the ammonite biozones, and belemnite palaeotemperatures. *Earth and Planetary Science Letters*, 179, 269-285 (2000).

35. Hesselbo, S. P., Jenkyns, H., Duarte, L., Oliveira, L. C. Carbon-isotope record of the Early Jurassic (Toarcian) Oceanic Anoxic Event from fossil wood and marine carbonate (Lusitanian Basin, Portugal). *Earth and Planetary Science Letters*, 253, 455–470 (2007).

36. Hesselbo, S. P., *et al*. Massive dissociation of gas hydrate during a Jurassic oceanic anoxic event. *Nature*, 406, 392-395 (2000).

37. Xu, W. *et al*. Evolution of the Toarcian (Early Jurassic) carbon-cycle and global climatic controls on local sedimentary processes (Cardigan Bay Basin, UK). *Earth and Planetary Science Letters*, 484, 396-411 (2018).

38. McElwain, J. C., Wade-Murphy, J., Hesselbo, S. P. Changes in carbon dioxide during an oceanic anoxic event linked to intrusion into Gondwana coals. *Nature*, 435, 479–482 (2005).

39. Drever, J. I. The Geochemistry of Natural Waters, *Englewood Cliffs: Prentice hall* (third edition), 436 (1997).

40. Freeman, K. H., & Hayes, J. M. Fractionation of carbon isotopes by phytoplankton and estimates of ancient CO_2 levels. *Global Biogeochemical Cycles*, 6, 629-644 (1992).
